# How effective are interventions to reduce damage to agricultural crops from herbivorous wild birds and mammals? A systematic review protocol

**DOI:** 10.1186/s13750-023-00315-0

**Published:** 2023-11-01

**Authors:** Ann Eklund, Johan Månsson, Jens Frank

**Affiliations:** Grimsö Wildlife Research Station, Department of Ecology, Swedish University of Agricultural Sciences, 730 91 Riddarhyttan, Sweden

**Keywords:** Wildlife damage prevention, Wildlife impact mitigation, Human wildlife conflict, Crop damage, Herbivore, Systematic review

## Abstract

**Background:**

An important conservation challenge is to mitigate negative impacts that wild birds and mammals can have on human practices and livelihoods, and not least on agricultural crops. Technical interventions to limit the number and severity of damages are available, but evaluations of intervention effectiveness are usually limited in scope, and meta-analyses are rare. This protocol describes a systematic review that seeks to answer the following question: How effective are evaluated interventions in reducing damage from herbivorous wild birds and mammals on agricultural crops?

**Methods:**

The literature searches are made in the databases Scopus and Zoological Record. The search string is based on a Population-Intervention-Comparator-Outcome (PICO) formatted research question, and search terms fall within five categories: Wildlife type (Population), Damage object (Population), Counteraction (Intervention), Evaluation (Comparator), and Damage (Outcome). Initial scoping searches informed amendment of the search string. A set of 19 benchmark articles were used to estimate the ability of the scoping search to capture relevant literature. To be eligible for inclusion in the review, original articles should study cases where settings of exposure to interventions (measures implemented to reduce damages on agricultural crops caused by terrestrial birds and mammals) are compared to a control setting without exposure to interventions. Eligible studies will be subject to data extraction, systematically documented in an Excel spreadsheet. Associated risk of bias will be critically appraised for the included articles according to seven criteria: 1. risk of confounding biases, 2. risk of post-intervention selection biases, 3. risk of misclassified comparison biases (observational studies only), 4. risk of performance biases (experimental studies only), 5. risk of detection biases, 6. risk of outcome reporting biases, and 7. risk of outcome assessment biases. The results will be reported in narrative and, if possible, quantitative syntheses. The quantitative synthesis will include a summary statistic calculated based on the data of each study and illustrated graphically in a forest plot. If possible, meta-regression analyses will be conducted.

**Supplementary Information:**

The online version contains supplementary material available at 10.1186/s13750-023-00315-0.

## Background

In response to global biodiversity loss, conservation science and practice is actively seeking to improve the status of native wildlife (in our context, wild animal species) populations under threat, often dependent on land sharing with other human interests and practices in multiuse landscapes [1, 2]. One of the main conservation challenges when humans and wildlife co-occur is the mitigation of negative impacts that wildlife can have on human property, referred to as wildlife damage [3–5]. Wildlife damage, often captured under the umbrella-term Human-Wildlife Conflicts (HWC), occurs on all continents with permanent human settlements and involves wildlife species of conservation concern that have negative impacts on human interests in both marine and terrestrial environments [6, 7]. In the terrestrial context impacts include damage caused by birds and mammals (such as elephants, primates, wild boar, geese) on agricultural crops, which is the basis for human food production [6]. Only in Sweden, the estimated amount of grain lost to wildlife reached 165,000 tons in 2020 [8]. Such crop damage is a concern for affected farmers and producers but may also threaten co-occurrence with species of high conservation concern if tolerance for the species is reduced with higher levels of damage.

Consequently, conservation and management of wild birds and mammals often focus on developing and implementing practical and technical interventions to limit the amount and severity of damage, not least on agricultural crops [9]. Among interventions there are examples of physical and psychological barriers such as fences and walls (e.g., [10, 11]), deterrents based on aversive auditory, olfactory, visual, or tactile stimuli to repel wildlife (e.g., [12, 13]); scaring actions and devices that induce flight response in wildlife (e.g., [14–16], or removal of wildlife from particularly problematic areas through hunting or translocation (e.g., [15, 17, 18]. Due to physiological, psychological, and behavioral traits characteristic of vertebrate species, that differ compared to for instance insects, we focus only on birds and mammals in relation to different wildlife management or damage interventions in this protocol. Henceforth we refer to these taxonomic groups as “wildlife”.

Evaluations of interventions’ effectiveness are usually limited to specific wildlife species, taxonomic groups, or geographical regions (e.g., [15, 19]) and meta-analyses are rare [3, 20]. Although the effect of interventions can be species or situation specific, it is also possible that management practices for one species or situation can draw learnings from other contexts. Furthermore, stakeholders and wildlife managers may face situations where multiple species cause damage, requiring multi-species assessment and a toolbox to handle complex situations (e.g., [21, 22]. Literature review and synthesis focusing on intervention effectiveness across taxonomic groups and geographical regions would thereby provide a useful overview of the current scientific knowledge, and guide future research towards current knowledge gaps. The protocol is registered in PROCEED (https://www.proceedevidence.info/site/index), manuscript number PROCEED-23–00167. Together with the supplementary ROSES form (Additional file 1, [23]), it describes the undertaking of a systematic review of published evidence for intervention effectiveness to protect agricultural crops from damage caused by native wild birds and mammals. Because native wildlife is a conservation priority, interventions to mitigate its impacts on, for instance agricultural crops is a priority to reduce conservation conflicts [24]. Introduced species, particularly those that have become invasive, do not generally have an important ecosystem function and therefore their management differs [1]. The aim of the systematic review and synthesis is to provide wildlife managers, conservationists, and agricultural practitioners with easily accessed knowledge of available interventions and their effectiveness.

The review is commissioned by the Swedish Wildlife Damage Centre (SWDC) on behalf of their funders, to update the website EviWild with syntheses of scientific evidence. The SWDC works closely with wildlife managers and practitioners, from whom they collate and receive feedback about the EviWild website, to ensure it develops as a useful tool for these practitioners. The SWDC representatives have been involved in the formulation of the research questions and setting the scope and focus of the review. They have been actively involved in the initial development of the search strategy and search string. Additionally, they provide input and set the direction for the review, to meet their output needs as defined by their funders. These stakeholders are, and will continue to be, engaged as co-authors on the project. Through written communication alongside multiple workgroup meetings, they will also be able to provide feedback on all parts of the review process.

## Objective of the review

The review seeks to answer the following question:

How effective are evaluated interventions in reducing damage from herbivorous wild birds and mammals on agricultural crops?

## Methods

### Searching for articles

The literature searches will be made according to the subscriptions of the Swedish University of Agricultural Sciences in Scopus and Zoological Record. Searches will be made in titles, abstracts, and keywords of articles in Scopus (TITLE-ABS-KEY). In Zoological Record publications are searched using topic terms (field tag: TS =) which include titles and abstracts alongside for instance descriptors and organism details. Searches in Zoological Record are undertaken with the Web of Science search engine, using the exact search option. No date or language restrictions will be applied during the search, although inclusion of studies in the analysis will be restricted to English and Swedish language due to the language limitations of the review team. Searches in Scopus will be limited to the two subject areas “Agricultural and Biological Sciences” and “Environmental Science”.

In the development of the search strategy the work group, comprised by the review team and an additional member of the SWDC, initially identified search term categories corresponding to the P-I-C-O elements (described in detail in the *Eligibility Criteria* section) in brackets: Wildlife type (Population), Damage object (Population), Counteraction (Intervention), Evaluation (Comparator), and Damage (Outcome). From the initial search terms identified by the work group within these categories, scoping searches were made in Web of Science (all databases), and additional search terms were added to the categories based on this initial search. A set of 19 benchmark articles (Additional file 2) were used to estimate the ability of the search string to return the relevant articles. Five of these benchmark articles were added by the work group [14, 15, 25–27]. An additional 14 benchmark articles were identified through the online library collated by the IUCN work group on Human-Wildlife Conflict and Coexistence (HWC). The main author manually screened all the references listed under the topics “Deterrents & repellents”, “Electric fences”, and “Other barriers” as these were the most relevant topics for the review research question. Eligible references were added to the list of benchmark articles (Additional file 2).

Further scoping searches based on the original search terms returned 42 and 53 percent of the benchmark articles in Scopus and Zoological Record. Fewer benchmark articles were returned, and no benchmark articles were exclusively returned, from scoping searches in Web of Science Core Collection, BIOSIS Citation Index, and CABI: CAB Abstracts ®. Therefore, Scopus and Zoological Record were determined the most relevant databases for this review. Informed by the missing benchmark articles additional search terms were added to each search category. Population and intervention terms were initially broad (e.g., wildlife, bird, mammal, protect, mitigate), whereas benchmark articles were sometimes very specific in their titles, abstracts, and keywords (e.g., goose, elephant). To determine the relevant species to include in the search, a prior review of species involved in HWC and also specifically crop damage, was consulted [28]. Common names of the species were added to the Wildlife search term category. For groups of species the group part of the common name was used as the search term, e.g., “geese” or “elephants”, rather than the species common name, e.g., “Canada geese” or “Asian elephants”. Additional intervention terms were added, and amendments were made to the string so that finally 100% of the benchmark articles were returned by the two databases. The final search terms and search strings are presented in full in Additional file 3.

In addition to searching the two databases using the complete search string, manual searches will be undertaken to capture grey literature. Agricultural organizations are expected to evaluate interventions to prevent damage to crops, and searches for research will therefore be undertaken on main organizational websites. This involves screening research on the *Environment* topic, and sub-topic *Wildlife, animals, biodiversity and ecosystems* on the website of the UK government (https://www.gov.uk/search/research-and-statistics), and using population terms from the search string to search for relevant titles in the catalogue of the Foods and Agriculture Organization of the United Nations (FAO, https://www.fao.org/library/libraryhome/en/), the USDA Economics, Statistics and Market Information System (ESMIS) developed and maintained by Mann Library at Cornell University on behalf of the United States Department of Agriculture (USDA, https://usda.library.cornell.edu/?locale=en), and the online Joint Research Centre Publications Repository of the European Commission (https://publications.jrc.ec.europa.eu/repository/).

Any review papers returned by the searches will not be included in the syntheses and analyses of the current review, but original articles included in the reference lists provided by such reviews will be screened. Furthermore, after screening of all returned articles, reference lists of the final set of articles for analysis will also be searched for relevant articles. Using the citation search in Web of Science, articles which have cited the final set of articles will also be subject to eligibility screening. A search update may be undertaken before the study is finalized but is subject to time and budget limitations.

### Article screening and study eligibility

#### Screening process

Scoping searches indicate an expected return of approximately 10,700 titles from Scopus and 8,700 titles from Zoological Record. All titles and abstracts will be imported to an online Rayyan (https://www.rayyan.ai/) account, in which exact duplicates will be automatically removed. The “detect duplicates” function will be used to identify other possible duplicate articles which will be checked manually, and if confirmed as duplicates, will be deleted. The subsequent manual screening of the returned literature will be undertaken in two steps. In the first step all titles and abstracts will be manually screened for inclusion of an eligible population and intervention according to the review eligibility criteria. The first screening step will be undertaken in Rayyan. The expected number of articles (~ 30 K) exceeds the resources for including two reviewers of all articles. Therefore, one reviewer will screen all the titles/abstracts (except any articles authored by the reviewer her/himself) and a random sample comprised of five percent of the titles/abstracts will be manually screened by two reviewers. Cohen’s Kappa will be calculated to estimate consistency between reviewers. Any inconsistencies will be discussed between screeners and resolved. Articles which are assessed as clearly irrelevant in the first step will be removed. Relevant articles and articles for which relevance cannot be determined by screening of titles and abstracts (because information provided in titles and abstracts is insufficient to assess eligibility, or because the article lacks an abstract), will be retained for the second screening step.

In this second step, articles will be subject to a full-text reading and assessed according to all eligibility criteria of the review. The eligibility screening will be recorded in an Excel spreadsheet (Additional file 4) under the ELIGIBILITY tab where articles are provided with a article ID number, and bibliographic information (title, author, publication year, journal) will be extracted. Eligibility for each criterion (population, intervention, comparator, outcome) is coded: yes/no/unclear. Articles which meet all eligibility criteria (coded: yes) will be included in the analysis and synthesis, while articles that fail to meet one or several of the eligibility criteria (coded: no) will not. In cases where insufficient information to assess eligibility is available from the full-text reading, more information will be sought from the authors of the article. Records of all communications with the authors, including the date when contact is made (in either direction) and what is communicated, will be recorded in a Word document together with the article ID number (Additional file 5). If article eligibility in one or several criteria remains unclear (coded: unclear) at this point, the article will not be included in the further analysis and synthesis but will remain listed in the datasheet.

If suspectedly linked articles (i.e., articles based on the same study or data collection) are detected during the full text reading, it will be noted in the ELIGIBILITY datasheet. Authors of the original papers will be contacted to dismiss or verify linkages if the link is uncertain from reading the articles. All communications with the authors, are recorded in the author communications document (Additional file 5). Where linkages remain uncertain, for instance if authors do not respond, the suspected linkages will be reported in the synthesis of the review, alongside reporting of verified linkages.

Eligibility screening from full-text reads is undertaken by one reviewer, with a random sample of a minimum of five percent that is screened by two reviewers. Cohen’s Kappa coefficient will be calculated to evaluate consistency between reviewers. Where reviewers disagree on their decisions, they will meet to discuss the decisions until consensus is reached. If the discrepancy cannot be solved by the reviewers themselves, other members of the review team will be consulted. Different members of the review team will be included as “double reviewers” to detect any potential systematic error of the main reviewer. To ensure independence of the reviews, any member of the review team who appears as an author on an included study will not be involved in evaluating the eligibility of the paper or in judging its validity.

#### Eligibility criteria

To be eligible for inclusion in the review, articles must meet the Population, Intervention, Comparator, and Outcome (PICO) elements underlying the review research question. The question and the criteria were developed together with the stakeholders to meet their needs. Benchmark articles 1–10 were subjected to eligibility screening with two reviewers to assess the formulation of the eligibility criteria. Articles included in the analysis will report studies with the following elements:

1. **Population.** Agricultural crops, i.e., any plants or plant parts intentionally grown by people for food consumption of people or domestic animals, under threat from terrestrial herbivorous wildlife. In the review context, terrestrial herbivorous wildlife is defined as wild (i.e., not domesticated) birds and mammals of regionally or migratory native species (i.e., not introduced, feral, or invasive species), that are free-living in the wild (i.e., not captive or tamed animals).
2. **Intervention.** Any method, action, or technology implemented to reduce the negative impact (e.g., damage, depredation, destruction) of terrestrial herbivorous wildlife on growing agricultural crops.
3. **Comparator.** Treatment (exposure to the evaluated intervention) setting compared to a control (no exposure to the evaluated intervention) setting. Apart from the exposure to the evaluated intervention in the treatment setting, all else should be equal (i.e., control settings will not be exposed to another intervention unless the treatment setting is also exposed to the same).
4. **Outcome.** Quantitative measures and comparisons of the occurrence or severity of wildlife damage on agricultural crops in the treatment and control settings, i.e., evaluations of intervention effectiveness.

Furthermore, to be included in the review articles should be written in English or Swedish, due to the language limitations of the review team. Original studies in different types of articles (e.g., journal articles, book chapters, proceedings, reports etc.) will be considered for the review. Previous review papers will not be included in the analysis. A list of all articles excluded at full-text reading, and the reason for their exclusion, will be provided.

### Study validity assessment

In the critical appraisal of study validity, at least two reviewers will assess and judge each included study for its associated risk of bias. In uncertain cases the work group will be consulted. The judgments will be undertaken using the Collaboration for Environmental Evidence Critical Appraisal Tool prototype, version 0.3 [29]. The tool is developed for critical appraisal of studies within the field of environmental research, including applied ecology. Following the tool, risk of studies’ internal validity will be appraised according to seven criteria: 1. risk of confounding biases, 2. risk of post-intervention selection biases, 3. risk of misclassified comparison biases (observational studies only), 4. risk of performance biases (experimental studies only), 5. risk of detection biases, 6. risk of outcome reporting biases, and 7. risk of outcome assessment biases [29]. To make critical appraisal as transparent as possible, judgments for each critical appraisal question of the CEE critical appraisal tool [29] will be listed for all the studies in a decisions sheet. The overall bias judgments for each study will also be recorded in the data sheet (Additional file 4

) and presented for all included studies in a table, which also includes a concise textual justification for the decisions.

### Data coding and extraction strategy

Data coding and extraction undertaken for studies eligible for the review analysis will mainly be undertaken by one reviewer, with a minimum of 5% of the studies subjected to double review for consistency checking. Where disagreements occur, these will be discussed between the reviewers until consensus is reached, or if the disagreement is not resolved then the work group will be consulted to reach consensus. Data extraction and coding will be undertaken in the review data sheet (Additional file 4), under the tab “ANALYSIS”. The data sheet was developed in a pilot test of benchmark articles 1–10, and two reviewers. We will extract and code data to map the study context (e.g., geographic location, wildlife species, intervention category etc.), descriptives of the experiment (e.g., duration of study, statistical unit etc.), and data for effect estimates (e.g., sample size, effect measures). Detail of data coding and extraction is provided for each variable in the review data sheet (Additional file 4), under the tab “CODING INSTRUCTION”.

In studies where effect measures are reported in figures rather than text or tables, the estimates will be back transformed to numerical values using the online tool PlotDigitzer (https://plotdigitizer.com/app). Values will be extracted by two reviewers, and considering the potential challenges of using the software to ensure the estimations are valid and reliable [30]. In cases where data are missing in the original articles, the corresponding author of the study will be contacted via email, with a request to provide or confirm missing data. This will also be done in cases where we are unable to extract values from figures in the original article. All author communications are recorded in author communications document (Additional file 5). If authors do not respond, studies will be excluded from further analysis and the reason will be stated in the data extraction sheet. Extracted data records will be made available as additional files in the final review article.

### Potential effect modifiers/reasons for heterogeneity

The SWDC representatives were consulted to identify sources of heterogeneity, as experts on the topic. Because the review is taking a broad approach, intending to evaluate the effectiveness of interventions to prevent damage on various crops caused by multiple wildlife species and taxa globally, heterogeneity is expected among studies in the research designs as well as contextual effect modifiers. Wildlife species may be an effect modifier [9, 31] due to species specific behavioral patterns or physical adaptations, corresponding to “clinical diversity” in medical trials [32]. For example, barriers such as fences may hinder mammals but not flying birds from entering an area. Care will be taken if studies evaluate intervention effectiveness in relation to different or mixed species or different crop types. Analyses and syntheses may be performed separately if species are observed effect modifiers. Other potential effect modifiers may relate to the biological factors (e.g., gender, age, or reproductive status) and behavior of individual animals but such effects may be difficult to identify in our analysis. The potential influence of individual trait effect modifiers may be discussed.

Effect modifiers may also relate to the physical context in which interventions are implemented or maintained. In prior studies we have identified discrepancies in the practical implementation and maintenance of interventions [19], and within intervention categories there may be different types of applications of an intervention, e.g., different types of fencing or scaring approaches [9, 31]. The interventions specifics are extracted in the data sheet, and discrepancies between models or designs may be observed. Implementation and maintenance needs will possibly vary in different settings and ecosystems, and care will be taken to observe potential effect modifiers related to intervention material and implementation.

### Data synthesis and presentation

The planned synthesis conducted as part of the systematic review will be narrative and, if possible, quantitative. Extracted and coded data will be made available in machine-readable and human-readable formats on publication of the final review. The narrative synthesis will be based on data extracted, and for each included study provide the article reference and describe in text the subject population (e.g., focal wildlife species, location of data collection), context (e.g., crop type, intervention material), methodological design and reported results of the study. Studies will be grouped and narratively presented according to the intervention type under investigation. The narrative synthesis will include a diagram and/or a table to visualize the results of each study and intervention type as well as provide a map of the geographic distribution of studies linked to intervention type. A diagram that illustrates the focal species for which the effectiveness of each intervention type has been evaluated, will also be included.

In the quantitative synthesis, a summary statistic (preferably logarithmic risk ratio) will be calculated based on the data of each study. The risk ratio is calculated as the ratio between the probability of yield loss (alternatively, proxies of the same e.g., wildlife abundance) in the treatment and the control setting. Because we are expecting that some studies will report dichotomous outcomes while others may report their results as continuous outcomes, recalculating the reported outcomes as risk ratios allows comparison between individual studies. In studies where the outcome is reported as count data, the data will be dichotomised prior to the calculation of risk ratio. In cases where effect estimates in the original studies are reported as continuous outcomes the conversion to a relative measure (such as the risk ratio) implies a loss of information [32]. Therefore, for studies reporting continuous outcomes, we will additionally calculate a standardized mean difference for comparisons. Meta-regression analysis will be undertaken in IBM SPSS software provided that the number of studies, and the data of the studies, comply with the assumptions of meta-regression. If meta-regression is not possible, then summary statistics for individual studies will be presented jointly (e.g., in tables and/or figures) based on their similarities (e.g., taxonomic, or physiological, traits of the wildlife involved and/or based on intervention sub-types) that provides some homogeneity of the data. Sensitivity analysis will be undertaken to identify potential variation in the overall effects when studies judged as having a high risk of bias are included or excluded from the analyses. The quantitative outcomes will be graphically presented in a forest plot, together with judgements of critical appraisals [32].

Many included articles are expected to be peer-reviewed, and thus an overall publication bias of the included studies may be suspected. Research protocols for the returned studies are expected to be missing, so to detect signs of publication bias, we seek to employ a funnel plot of the effect measure against the standard error of the effect measure for each study. If asymmetry is detected using the Egger test [33], then the review will discuss the possible underlying causes of the asymmetry, of which publication bias may be one cause [34]. Provided that grey literature is returned from the database search, or searches of organizational websites, the outcomes of these studies in relation to scientifically published papers, can inform the understanding of potential publication bias.

## Supplementary Information

**Additional file 1**. ROSES form.

**Additional file 2.** Benchmark articles.

**Additional file 3.**  Search string.

**Additional file 4.**  Data extraction sheet.

**Additional file 5.**  Author communications.

## Data Availability

All data generated or analysed during this study are included in the published article [and its additional information files].
