# Supplementary material for: How effective are interventions to reduce damage to agricultural crops from herbivorous wild birds and mammals? A systematic review protocol
Source: Environ Evid. 2023 Nov 1;12:22. doi: 10.1186/s13750-023-00315-0 (PMC11378812; doi:10.1186/s13750-023-00315-0)
Supplement: Supplementary file 1 — Additional file 1. ROSES form. [file 13750_2023_315_MOESM1_ESM.pdf]

Item number	Section / sub-section	Topic	Description	Further explanation	Checklist/Meta-data	Author response
1	Title	Title	The title must indicate that it is a systematic review protocol, and must	The title should normally be the same or very similar to the review	Meta-data	**How effective are interventions to reduce damage to agricultural crops from herbivorous wild birds and mammals? A Systematic Review Protocol**
2	Type of review	Type of review	Select one of the following types of review: systematic review, systematic	See CEE Guidance on amendments and updates [1]	Meta-data	systematic review
3	Authors contacts	Authors contacts	The full names, institutional addresses, and email addresses for all		Checklist	Yes
4	Abstract	Structured summary	Abstract must not exceed 350 words and must include two sections 1)		Checklist	Yes
5	Background	Background	Describe the rationale for the review in the context of what is already	A theory of change and/or conceptual model can be presented that links	Checklist	Yes
6	Stakeholder engagement	Stakeholder engagement	The planned/actual role of stakeholders throughout the review process		Checklist	Yes
7	Objective of the review	Objective	Describe the primary question and secondary questions (when	The primary question is the main question of the review. Secondary	Checklist	Yes
8		Definitions of the question	Break down and summarise question key elements e.g. population,	For other question types see [3,4]	Meta-data	Population: Terrestrial herbivorous wildlife, defined as wild (i.e., not domesticated) birds and mammals of regionally or migratory native species (i.e., not introduced, feral, or invasive species), that are free-living in the wild (i.e., not captive or
	Methods					
9	Searches	Search strategy	Detail the planned search strategy to be used, including: database names	Details regarding search strategy testing should be provided.	Checklist	Yes
10		Search string	Provide Boolean-style full search string and state the platform for which		Meta-data	WoS: (TS= ((lark* OR mallard* OR goose OR geese OR swan* OR waxwing* OR hornbill* OR * bananaquit* OR crow* OR raven* OR crane* OR blackbird* OR “black bird*” OR cowbird* OR grackle* OR bee-eater* OR guineafowl OR francolin* C
11		Languages – bibliographic	List languages to be used in bibliographic database searches.		Meta-data	English and Swedish
12		Languages – grey literature	List languages to be used in organizational websites searches and web-		Meta-data	English and Swedish
13		Bibliographic databases	Provide the number of bibliographic databases to be searched.		Meta-data	Zoological Record, Scopus
14		Web – based search engines	Provide the number of web – based search engines to be searched.		Meta-data	N/A
15		Organisational websites	Provide the number of organisational websites to be searched.		Meta-data	https://www.gov.uk/search/research-and-statistics, https://www.fao.org/library/libraryhome/en/, https://usda.library.cornell.edu/?locale=en, https://publications.jrc.ec.europa.eu/repository/
16		Estimating the comprehensiveness	Describe the process by which the comprehensiveness of the search		Checklist	Yes
17		Search update	Describe any plans to update the searches during the conduct of the	Optional. A search update is good practice if original searches were	Checklist	Yes
18	Article screening and study	Screening strategy	Describe the methodology for screening articles/studies for		Checklist	Yes
19		Consistency checking	Describe clearly the process for checking consistency of decisions		Checklist	Yes
20		Inclusion criteria	Describe the inclusion criteria used to assess relevance of identified		Checklist	Yes
21		Reasons for exclusion	State that you will provide a list of articles excluded at full text with		Checklist	Yes
22	Critical appraisal	Critical appraisal	Describe here the method you propose for critical appraisal of study		Checklist	Yes
23		Critical appraisal strategy	Describe how the information from critical appraisal will be used in		Checklist	Yes
24		Consistency checking	Describe how repeatability of critical appraisal of study validity will be		Checklist	Yes
25	Data extraction	Meta-data extraction and coding	Describe the method for meta-data extraction and coding for studies		Checklist	Yes
26		Data extraction strategy	Describe the method for extraction of qualitative and/or quantitative		Checklist	Yes
27		Approaches to missing data	Describe any processes for obtaining and confirming missing or unclear		Checklist	Yes
28		Consistency checking	Describe how repeatability of the meta-data/data extraction process will		Checklist	Yes
29	Potential effect modifiers/reasons	Potential effect modifiers/reasons	Provide a list of and justification for the effect modifiers /reasons for	The list should not be exhaustive but a short list of those variables	Checklist	Yes
30	Data synthesis and presentation	Data synthesis and presentation	State the type of synthesis conducted as part of the systematic review		Meta-data	Narrative and Quantitative
31		Narrative synthesis strategy	Describe methods to be used for narratively synthesising the evidence	Vote-counting (tallying of studies based on the direction or significance	Checklist	Yes
32		Quantitative synthesis strategy	If data are appropriate for quantitative synthesis, describe planned	Compulsory if appropriate for data	Checklist	Yes
33		Qualitative synthesis strategy	Describe methods to be used for synthesising qualitative data and justify	Compulsory if appropriate for data	Checklist	Yes
34		Other synthesis strategies	Describe any other approaches to be used for synthesising data or	Compulsory if appropriate for data	Checklist	Yes
35		Assessment of risk of publication	Describe planned methods for examining the possible influence of	For quantitative syntheses this may be done using diagnostic plots or	Checklist	Yes
36		Knowledge gap identification	Describe the methods to be used to identify and/or prioritise key	Optional	Checklist	n/a
37		Demonstrating procedural	Describe the role of systematic reviewers (who have also authored	Reviewers who have authored articles to be considered within the review	Checklist	Yes
38	Declarations	Competing interests	Describe of any financial or non-financial competing interests that the		Checklist	Yes

References

[1] Bayliss, H.R., Haddaway, N.R., Eales, J., Frampton, G.K. and James, K.L., 2016. Updating and amending systematic reviews and systematic maps in environmental management. Environmental Evidence, 5(1), p.20.

[2] Haddaway, N.R., Kohl, C., da Silva, N.R., Schiemann, J., Spök, A., Stewart, R., Sweet, J.B. and Wilhelm, R., 2017. A framework for stakeholder engagement during systematic reviews and maps in environmental management. *Environmental Evidence* , *6* (1), p.11.

[3] Collaboration for Environmental Evidence. 2018. Guidelines and Standards for Evidence synthesis in Environmental Management. Version 5.0. www.environmentalevidence.org/information-for-authors.

[4] Leeds Institute of Health Sciences. https://medhealth.leeds.ac.uk/info/639/information_specialists/1500/search_concept_tools. Accessed 12/11/2017.
