# Supplementary material for: How effective are interventions to reduce damage to agricultural crops from herbivorous wild birds and mammals? A systematic review protocol
Source: Environ Evid. 2023 Nov 1;12:22. doi: 10.1186/s13750-023-00315-0 (PMC11378812; doi:10.1186/s13750-023-00315-0)
Supplement: Supplementary file 2 — Additional file 2. Benchmark articles. [file 13750_2023_315_MOESM2_ESM.docx]

**Benchmark articles**

References derived from the review work group:

1. Clausen KK, Marcussen LK, Knudsen N, Balsby TJS, Madsen J. 2019. Effectiveness of lasers to reduce goose grazing on agricultural grassland. Wildlife Biology, DOI: 10.2981/wlb.00560.
2. Heim W, Piironen A, Heim RJ, Piha M, Seimola T, Forsman JT, Laaksonen T. 2022. Effects of multiple targeted repelling measures on the behaviour of individually tracked birds in an area of increasing human-wildlife conflict. Journal of Applied Ecology, DOI: 10.1111/1365-2664.14297.
3. Månsson J. 2017. Lethal scaring e Behavioral and short-term numerical response of greylag goose Anser anser. Crop Protection, 96: 258-264.
4. Summers RW, Hillman G. 1990. Scaring brent geese Branta bernicla from fields of winter wheat with tape. Crop protection, 9: 459-462.
5. Tombre IM, Madsen J, Tømmervik H, Haugen T-P, Eythórsson E. 2005. Influence of organized scaring on distribution and habitat choice of geese on pastures in Northern Norway. Agriculture, Ecosystems & Environment, 111: 311-320.

References derived from the IUCN Human Wildlife Conflict (HWC) specialist group:

1. Adams, T. S. F., I. Mwezi, and N. R. Jordan. 2020 (2021). Panic at the disco: solar-powered strobe light barriers reduce field incursion by African elephants Loxodonta africana in Chobe District, Botswana. Oryx:1-8. External resource
2. Campbell-Smith, G., R. Sembiring, and M. Linkie. 2012. Evaluating the effectiveness of human-orangutan conflict mitigation strategies in Sumatra. Journal of Applied Ecology 49:367-375. External resource
3. Dieter, C. D., C. S. Warner, and C. Ren. 2014. Evaluation of foliar sprays to reduce crop damage by Canada geese. Human-Wildlife Interactions 8:139-149. Download
4. González, L. M., F. G. D. Montoto, T. Mereck, J. Alves, J. Pereira, P. F. de Larrinoa, A. Maroto, L. Bolonio, and N. El-Kadhir. 2017. Preventing crop raiding by the vulnerable common hippopotamus Hippopotamus amphibius in Guinea-Bissau. Oryx 51:222-229. External resource
5. Graham, M. D., and T. Ochieng. 2008. Uptake and performance of farm-based measures for reducing crop raiding by elephants Loxodonta africana among smallholder farms in Laikipia District, Kenya. Oryx 42:76-82. External resource
6. Hedges, S., and D. Giunaryadi. 2010. Reducing human-elephant conflict: do chillies help deter elephants from entering crop fields? Oryx 44:139-146. External resource
7. King, L. E., I. Douglas-Hamilton, and F. Vollrath. 2011. Beehive fences as effective deterrents for crop-raiding elephants: field trials in northern Kenya. African Journal of Ecology 49:431-439. Download
8. King, L. E., A. Lawrence, I. Douglas-Hamilton, and F. Vollrath. 2009. Beehive fence deters crop-raiding elephants. African Journal of Ecology 47:131-137. External resource
9. Laguna, E., P. Palencia, A. J. Carpio, J. Mateos-Aparicio, C. Herraiz, C. Notario, J. Vicente, V. Montoro, and P. Acevedo. 2022. Evaluation of a combined and portable light-ultrasound device with which to deter red deer. European Journal of Wildlife Research 68:50. Download
10. Osborn, F. V. 2002. Capsicum oleoresin as an elephant repellent: field trials in the communal lands of Zimbabwe. Journal of Wildlife Management 66:674-677. External resource
11. Pozo, R. A., T. Coulson, G. McCulloch, A. Stronza, and A. Songhurst. 2017 (2019). Chilli-briquettes modify the temporal behaviour of elephants, but not their numbers. Oryx:1-9. External resource
12. Sitati, N. W., and M. J. Walpole. 2006. Assessing farm-based measures for mitigating human-elephant conflict in Transmara District, Kenya. Oryx 40:279-286. External resource
13. Widén, A., M. Clinchy, A. M. Felton, T. R. Hofmeester, D. P. J. Kuijper, N. J. Singh, F. Widemo, L. Y. Zanette, and J. P. G. M. Cromsigt. 2022. Playbacks of predator vocalizations reduce crop damage by ungulates. Agriculture, Ecosystems & Environment 328:107853. Download
14. Kioko, J., P. Muruthi, P. Omondi, and P. I. Chiyo. 2008. The performance of electric fences as elephant barriers in Amboseli, Kenya. South African Journal of Wildlife Research 38:52-58. External resource
