# Supplementary material for: How effective are interventions to reduce damage to agricultural crops from herbivorous wild birds and mammals? A systematic review protocol
Source: Environ Evid. 2023 Nov 1;12:22. doi: 10.1186/s13750-023-00315-0 (PMC11378812; doi:10.1186/s13750-023-00315-0)
Supplement: Supplementary file 3 — Additional file 3. Search string. [file 13750_2023_315_MOESM3_ESM.docx]

**Search string**

Search terms, search categories, and search strings for Scopus and Zoological Record. The wildcard * represent any number of characters or zero, to allow searches for different variations of terms. In Web of Science “___” are used for searching exact terms, and in Scopus exact terms are searched using {___}.

| *Population* | | *Population* | *Outcome* | *Intervention* | *Comparator* |  |
| --- | --- | --- | --- | --- | --- | --- |
| **WILDLIFE** | | **DAMAGE OBJECT** | **DAMAGE** | **COUNTER-ACTION** | **EVALUATION** |  |
| lark* mallard* goose geese swan* waxwing* hornbill*  bananaquit*  crow*  raven* crane* blackbird* “black bird*”  cowbird* grackle*  bee-eater* guineafowl francolin* turkey* flamingo*  woodpecker* parrot* parakeet* ringneck* cockatoo* corella* galah* bonnet*  rosella* bulbul* starling* robin*  impala* blackbuck* bison* gaur* nilgai* buffalo* ibex duiker* wildebeest topi* gazelle* antelope*  waterbuck* “buffon’s kob” dik-dik* | bushbuck* sitatunga* kudu* racoon*  capuchin* monkey* mangabey* guenon* colobus*  macaque* baboon* langur* moose* chital* deer elk muntjac*  vole* agouti* elephant* zebra* giraffe* mongoose* hippopotamus gorilla*  chimpanzee*  orangutan*  porcupine* hare*  rabbit* rodent* mouse  mice marten* badger* civet* hyrax “fruit bat*” squirrel* warthog* bushpig*  “buch pig*” “warty pig*” “wild boar*” peccaries  “cane rat*” | crop* fruit* vegetable* orchard* lettuce plant* cultivar* grain* seed* pasture* field* farmland* cropland* grassland* ley “arable land” | damage* strike* raid* depredat* destroy destruction impact* loss | protect* prevent* mitigat* intervention* action* repel* scare scaring displace displacing divert* deter* “supplementary feeding” barrier* hunt* fence* spray* | trial* experiment* evaluat* effect* |  |
| *Scopus:*  TITLE-ABS-KEY(lark* OR mallard* OR goose OR geese OR swan* OR waxwing* OR hornbill* OR * bananaquit* OR crow* OR raven* OR crane* OR blackbird* OR {black bird*} OR cowbird* OR grackle* OR bee-eater* OR guineafowl OR francolin* OR turkey* OR flamingo* OR woodpecker* OR parrot* OR parakeet* OR ringneck* OR cockatoo* OR corella* OR galah* OR bonnet* OR rosella* OR bulbul* OR starling* OR robin* OR impala* OR blackbuck* OR bison* OR gaur* OR nilgai* OR buffalo* OR ibex OR duiker* OR wildebeest OR topi* OR gazelle* OR antelope* OR waterbuck* OR {buffon’s kob} OR dik-dik* OR bushbuck* OR sitatunga* OR kudu* OR racoon* OR capuchin* OR monkey* OR mangabey* OR guenon* OR colobus* OR macaque* OR baboon* OR langur* OR moose* OR chital* OR deer OR elk OR muntjac* OR vole* OR agouti* OR elephant* OR zebra* OR giraffe* OR mongoose* OR hippopotamus OR gorilla* OR chimpanzee* OR orangutan* OR porcupine* OR hare* OR rabbit* OR rodent* OR mouse OR mice OR marten* OR badger* OR civet* OR hyrax OR {fruit bat*} OR squirrel* OR warthog* OR bushpig* OR {buch pig*} OR {warty pig*} OR {wild boar*} OR peccaries OR {cane rat*} AND crop* OR fruit* OR vegetable* OR orchard* OR lettuce OR plant* OR cultivar* OR grain* OR seed* OR pasture* OR field* OR farmland* OR cropland* OR grassland* OR ley OR {arable land} AND damage* OR strike* OR raid* OR depredat* OR destroy OR destruction OR impact* OR loss AND protect* OR prevent* OR mitigat* OR intervention* OR action* OR repel* OR scare OR scaring OR displace OR displacing OR divert* OR deter* OR {supplementary feeding} OR barrier* OR hunt* OR fence* OR spray* AND trial* OR experiment* OR evaluat* OR effect* ) | | | | | | |
| *Zoological Records:* (TS= ((lark* OR mallard* OR goose OR geese OR swan* OR waxwing* OR hornbill* OR * bananaquit* OR crow* OR raven* OR crane* OR blackbird* OR “black bird*” OR cowbird* OR grackle* OR bee-eater* OR guineafowl OR francolin* OR turkey* OR flamingo* OR woodpecker* OR parrot* OR parakeet* OR ringneck* OR cockatoo* OR corella* OR galah* OR bonnet* OR rosella* OR bulbul* OR starling* OR robin* OR impala* OR blackbuck* OR bison* OR gaur* OR nilgai* OR buffalo* OR ibex OR duiker* OR wildebeest OR topi* OR gazelle* OR antelope* OR waterbuck* OR “buffon’s kob” OR dik-dik* OR bushbuck* OR sitatunga* OR kudu* OR racoon* OR capuchin* OR monkey* OR mangabey* OR guenon* OR colobus* OR macaque* OR baboon* OR langur* OR moose* OR chital* OR deer OR elk OR muntjac* OR vole* OR agouti* OR elephant* OR zebra* OR giraffe* OR mongoose* OR hippopotamus OR gorilla* OR chimpanzee* OR orangutan* OR porcupine* OR hare* OR rabbit* OR rodent* OR mouse OR mice OR marten* OR badger* OR civet* OR hyrax OR “fruit bat*” OR squirrel* OR warthog* OR bushpig* OR “buch pig*” OR “warty pig*” OR “wild boar*” OR peccaries OR “cane rat*”) AND (crop* OR fruit* OR vegetable* OR orchard* OR lettuce OR plant* OR cultivar* OR grain* OR seed* OR pasture* OR field* OR farmland* OR cropland* OR grassland* OR ley OR “arable land”) AND (damage* OR strike* OR raid* OR depredat* OR destroy OR destruction OR impact* OR loss) AND (protect* OR prevent* OR mitigat* OR intervention* OR action* OR repel* OR deter* OR scare OR scaring OR displace OR displacing OR divert* OR “supplementary feeding” OR barrier* OR hunt* OR fence* OR spray*) AND (trial* OR experiment* OR evaluat* OR effect*))) | | | | | | |
