# Supplementary material for: How effective are interventions to reduce damage to agricultural crops from herbivorous wild birds and mammals? A systematic review protocol
Source: Environ Evid. 2023 Nov 1;12:22. doi: 10.1186/s13750-023-00315-0 (PMC11378812; doi:10.1186/s13750-023-00315-0)
Supplement: Supplementary file 5 — Additional file 5. Author communications. [file 13750_2023_315_MOESM5_ESM.docx]

*Author Communications*

| **Study ID** | **Date** | **Communication** |
| --- | --- | --- |
|  |  |  |
|  |  |  |
|  |  |  |
|  |  |  |
|  |  |  |
|  |  |  |
|  |  |  |
|  |  |  |
|  |  |  |
|  |  |  |
|  |  |  |
|  |  |  |
|  |  |  |
|  |  |  |
|  |  |  |
|  |  |  |
|  |  |  |
|  |  |  |
